# Incidence of Dentinal Crack after Root Canal Preparation by ProTaper Universal, Neolix and SafeSider Systems

**DOI:** 10.22037/iej.v12i4.17597

**Published:** 2017

**Authors:** Azadeh Harandi, Sina Mirzaeerad, Mahgol Mehrabani, Elham Mahmoudi, Ali Bijani

**Affiliations:** a *Dental Materials Research Center, Institute of Health, Endodontic Department, Dental School, Babol University of Medical Sciences, Babol, IR. Iran; *; b * Student Research Committee, Dental School, Babol University of Medical Sciences, Babol, Iran*; c *Social Determinants of Health Research Center, Health Research Institute, Babol University of Medical Sciences, Babol, Iran *

**Keywords:** Dentinal Cracks, Micro Crack, Root Canal Preparation, Root Crack, Root Dentine, Single-file System

## Abstract

**Introduction:**

This study aimed to compare the incidence of dentinal crack formation by instrumentation with ProTaper Universal system (rotary, multi-file system), SafeSider (reciprocation movement, multi-file system) and Neolix (rotary, single-file system).

**Methods and Materials:**

In this *in vitro* study, 60 freshly extracted mandibular first molars were randomly divided into three experimental groups (*n*=15) and a control group containing unprepared teeth (*n*=15). Instrumentation in different groups was accomplished using either ProTaper, Neolix or SafeSider systems up to 25/0.08. The teeth were then sectioned at 3, 6 and 9 mm from the apex, and observed under a stereomicroscope for presence of dentinal cracks. Data were analyzed with *Chi* square test, Fisher’s exact test and Bonferroni correction.

**Results:**

Micro cracks were seen in all experimental groups (13.3% in ProTaper, 26.7% in SafeSider and 40% in Neolix). There was a significant difference between Neolix and the control groups in microcrack formation (*P*=0.042). Micro cracks mainly occurred in the coronal section (9 mm). No microcrack occurred in the control group.

**Conclusion:**

Neolix rotary single-file system caused more dentinal cracks compared to the unprepared roots. All the instrumentation systems increased the number of micro cracks compared to unprepared teeth.

## Introduction

Long-term success of root canal therapy highly depends on ideal biomechanical preparation of the canals [1]. The main goals of root canal instrumentation include complete elimination of bacteria, pulp tissue and debris and to prevent re-infection by proper obturation [2].

Various types of nickel-titanium (NiTi) files and rotary systems have been designed by the manufacturers in order to prevent the shortcomings of conventional files such as ledge formation, zipping and elbows formation [3]. Despite the advantages of NiTi instruments, such as increased flexibility, shorter working time and maintaining the natural canal curvature [4], serious problems may be caused by use of these instruments including dentinal micro crack formation and instrument fracture [5, 6]. NiTi rotary instruments have different tip designs, taper and cutting blade configuration and thus, stress concentration in dentinal walls may increase crack formation [7]. Different types of dentinal defects may occur such as craze lines, micro cracks or vertical root fracture (VRF). Accumulation of stresses during canal obturation and repeated occlusal forces can cause crack propagation into complete fracture [8]. VRF is a serious complication of endodontic procedures, which often necessitates tooth extraction [9]. Different instrumentation systems can cause various degrees of damage to the root canal wall [10, 11].

Root canal preparation with continuous rotary instruments has higher cutting efficiency but higher risk of instrument fracture due to higher level of torsion and flexion [12]. To avoid this, reciprocating movement was suggested that decreases the risk of instrument fracture by clockwise (cutting action) and counter clockwise (release of instrument) movements [13, 14]. It is claimed that preparation with reciprocating motion is the evolved version of the balanced force technique (16, 17). According to this claim, reciprocating motion may require less apical force for the advancement of instrument into the canal [15].

In the 1990s, several NiTi rotary instruments were introduced for more efficient endodontic treatment. ProTaper Universal was among these systems, with three shaping files [SX, auxiliary shaping file, tip size 17 to shape the coronal portion of the root canal, followed by S1 (tip size 20) in the coronal third and S2 (tip size 19) in the middle third][8]) and three finishing instruments [F1 (20/0.07), F2 (25/0.08) and F3 (30/0.09) and F4 (40/0.06)] [16].

SafeSider (Essential Dental Systems, South Hackensack, NJ, USA) system is a recently introduced reciprocating system. SafeSider files are reamers with approximately vertical flutes that give them the ability to remove more dentin. The system has 8 stainless-steel files (size ranges from 8 to 40) and three NiTi instruments and also a Pleezer for widening of the root canal. These reamers have sharp tips and their taper ranges from 2% to 8%.

Their stainless-steel material enables pre-curving of the file and their easier use in curved canals. Reamers larger than #15 are flat-sided. These instruments have reciprocating motion and they are being used with a special endodontic electric motor (30 degrees CW and 60 degrees CCW) [17].

Rotary systems can be categorized into single and multi-file systems. Preparation of the entire root canal by one single NiTi instrument has advantages such as being cost-effective, decreasing cross-contamination and reducing instrument fatigue [18].

Neoniti (NEOLIX, Châtres-la-Forêt, France) is among the single file systems. According to the manufacturer, rectangular non-similar cross-sections all along its length gives suitable flexibility to the instrument resulting in more efficient preparation of curved canals while preserving the initial anatomy of the root canal. This system has A1 and C1 files. C1 is used for opening and widening of the coronal portion of the canal (25/0.12 and 15 mm length). A1 file is used for preparing the apical portion and is produced with three different sizes (20/0.08, 25/0.08 and 40/0.08) that are recommended to be used with speed of 300 to 500 rpm and torque limit of 1.5 N/cm [19]. It may be assumed that using only one NiTi instrument for cleaning and shaping of the whole root canal space may increase stress concentration in the root canal walls compared to full-sequence systems and increase the risk of dentinal crack formation [9].This study aimed to compare dentinal crack formation by ProTaper Universal, Neolix and SafeSider systems.

## Materials and Methods

After ethics approval (MUBABOL.REC.1395.143), 60 freshly extracted mandibular first molars were selected for this *in vitro* study. The teeth were extracted for reasons not relevant to this research (periodontal or restorative reasons).

The teeth were cleaned with a periodontal scaler and stored in distilled water to prevent dehydration throughout the study. The teeth were then disinfected using 2.5% sodium hypochlorite and then observed with a stereomicroscope (Dewinter, Milano, Italy) under 25× magnification to evaluate the presence of micro cracks.

Radiographs were taken in buccolingual and mesiodistal directions from all teeth. Teeth with reduced pulpal space, pulp stones, calcified canals, hyper cementosis, root caries, internal or external root resorption, former root canal treatment, open apices and severely curved canals were excluded from this study and 60 teeth that met the inclusion criteria remained in the study.

Teeth included in this study were mandibular first molars with separate mesial and distal roots. After access cavity preparation with a diamond bur (Brasseler USA, Savannah, GA, USA), only teeth with moderate root curvatures (25-30^º^) [20] that had separate mesial canals (type III) were selected. 

The teeth were decoronated at the cementoenamel junction. Distal roots were also cut and only mesial roots with 13 mm length remained. Patency of the canals was maintained using ISO #10 K-file (Dentsply Maillefer, Ballaigues, Switzerland). The working length of each canal was determined by ISO #15 K-file (Dentsply Maillefer, Ballaigues, Switzerland) 1 mm short of the anatomic apex.

Cemental surfaces of the roots were coated with a thin layer of silicon impression material to simulate the periodontal ligament and were then mounted in acrylic resin to facilitate the next steps. Samples were divided into four groups and root canal preparation was accomplished as follows [21].

***Canal preparation***

Root canal instrumentation procedures were performed using three different systems namely ProTaper Universal, SafeSider and Neolix according to the manufacturers’ instructions. The teeth were prepared with a #25 master apical file in all systems.

**Figure 1:**
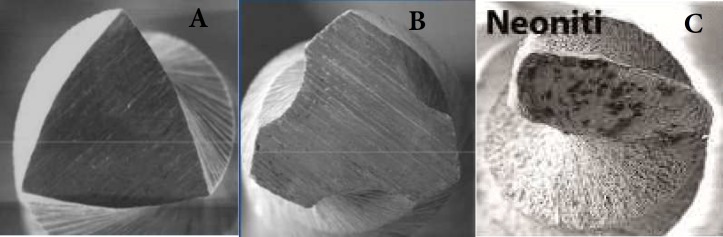
Cross sectional image of: *A)* ProTaper, *B)* SafeSider, *C)* Neolix instruments

Group 1 (*n*=15): This group served as the control group and the root canals remained unprepared in this group.

Group 2 (*n*=15): Root canals were prepared using ProTaper Universal (Dentsply Maillefer, Ballaigues, Switzerland) according to the manufacturer’s instructions. First, the canals were instrumented with S1 and S2 passively and then SX was used if necessary. Finally all the files (S1, S2, F1 and F2) were used to the same working length. Each file was reached to the working length in a passive manner (single-length manner). Instrumentation was done with the aid of an endodontic electric endomotor (Endo-Mate DT, NSK, Nakanishi Inc., Tokyo, Japan) operating at 300 rpm with a toque of 3 N/cm for shaping files, 1.5 N/cm for F1 and 2 N/cm for F2 file. Instrumentation was done with a light pecking in and out motion.

Group 3 (*n*=15): For cleaning and shaping of teeth in this group, SafeSider system (Essential Dental Systems, South Hackensack, NJ, USA) was used. Canal preparation was done with a sequence of #20 and 25 stainless-steel files (2% taper) to the same working length. Coronal enlargement was done with Pleezer and finally NiTi files # 25 (6% and 8% taper) were used for final preparation (6% taper for instrumenting the entire working length and 8% taper to prepare the root canal 2 mm short of the working length). Instrumentation was done using an Endo-Express reciprocating hand piece (reciprocation cycle: 1500-2000 rpm; Essential Dental Systems, Hackensack, NJ, USA). All these procedures were performed according to the manufacturer’s instructions. Instrumentation was done with a light pecking motion.

Group 4 (*n*=15): The root canals were prepared with Neolix single-file system. For enlargement of the coronal portion, C1 NiTi file (size 25/0.12) was used with brushing movement on safe canal walls. The apical two-thirds of the canal was prepared with A1 NiTi file (25/0.08) with brushing movement. According to the manufacturer, endodontic micromotor operating at 300 rpm and 1.5 Ncm torque was used. All instruments were dipped in RC prep before use to facilitate their movement and avoid fracture. Next, 2 mL of the freshly mixed 2% NaOCl was used for irrigation of each canal between the use of instruments using a syringe and a 27-gauge needle; 2 mL of distilled water was used for the final rinse of each canal. Each file was only used for instrumentation of one canal. 

***Sectioning and microscopic examination***

The roots were sectioned horizontally at 3, 6 and 9 mm distance from the apex with the aid of a low speed handpiece under water coolant (diamond disc’s thickness: 0.3 mm).

Sections (both mesial canals) were then observed under a stereomicroscope under 40× magnification. Digital images were captured with the aid of a digital camera attached to a stereomicroscope under 25× and 40× magnifications. Each sample was inspected by two experienced operators for presence of dentinal micro cracks. Disagreement between observers was resolved by discussing the case with a third experienced operator.

Data were divided into two groups of presence and absence of cracks. “Presence of cracks” was referred to the presence of any craze line, micro crack or fracture, and “absence of crack” was defined as roots with an intact dentin exempt of any craze line or micro crack. Craze lines were defined as dentinal defects that initiated from the external dentinal wall and did not involve the pulp space or internal dentinal wall. Micro cracks are defects that initiate from the internal wall and do not involve the external dentinal wall. Complete fractures are those involving both dentinal walls [8]. In each group, 45 sections were observed (a total of 180 slices). 

***Statistical analysis***

The results were expressed as the percentage and number of cracked roots in each group. The *Chi* square test, Fisher’s exact test and Bonferroni correction were used to compare micro crack formation among the groups. All the analyses were performed at a 95% confidence interval using SPSS software (SPSS version 17.0, SPSS, Chicago, IL, USA). The level of significance was set at 0.05.

## Results

There was a statistically significant difference between the Neolix group and the control group in terms of micro crack formation (*P*=0.033). Both rotary and reciprocating systems increased the number and percentage of cracks in roots (13.3% in ProTaper group, 26.7% in SafeSider group and 40% in Neolix group) (Table 1). But, there was no significant difference among the three groups (*P*>0.05). Micro cracks mainly occurred in the coronal section (9 mm), although was no statistically significant difference (*P*=0.486). No crack was observed in the control group (unprepared roots). Only one complete fracture was found in ProTaper group.

**Figure 2:**
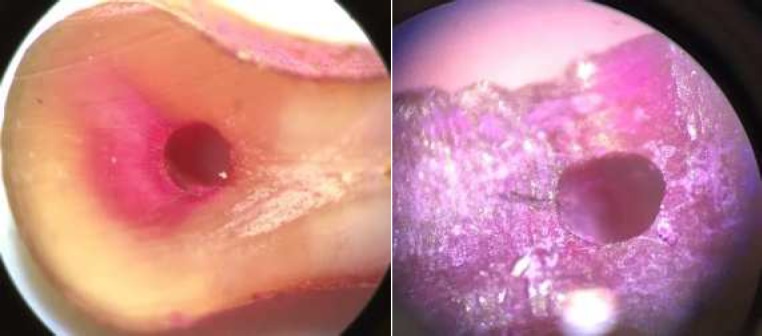
Samples instrumented with ProTaper system; *A)* Absence of crack, *B)* Presence of crack

## Discussion

In the present study, Neolix single-file rotary system showed significantly higher dentinal micro cracks compared to the control group. Since micro cracks were seen in all groups (except for the control group), none of the systems could prevent micro cracks. There was no statistically significant difference among the preparation groups (40% in Neolix, 26.7% in SafeSider and 13.3% in ProTaper groups). According to a study by De-Deus *et al.* [22], the sectioning method is a destructive procedure and can cause micro cracks. Shemesh *et al.* (8), and Bier *et al.* (10), also reported that dentinal micro cracks could occur during tooth extraction or sawing action. However, in our study, the control group did not show any micro cracks; therefore, we may conclude that the observed micro cracks were the result of the instrumentation 

process. Previous studies reported that single-file systems are four times faster than the conventional rotary systems for instrumentation [18, 23]. Higher number of micro cracks observed in the Neolix group may be the result of the sudden stress that is initially applied to dentinal walls. 

The mesial root of mandibular first molar has narrow canals. Introduction of #25 Neolix rotary file (with 0.08 taper) into a root canal with no prior instrumentation can cause heavy concentration of stress in dentinal walls. No previous study has been conducted regarding Neolix system according to our literature search. The results of our study were in agreement with those of Priya *et al.* [24], about single-file systems, who concluded that instrumentation with single-file systems caused more dentinal defects in comparison with full sequence systems. It may be the result of more stress concentration leading to micro crack formation.

**Figure 3:**
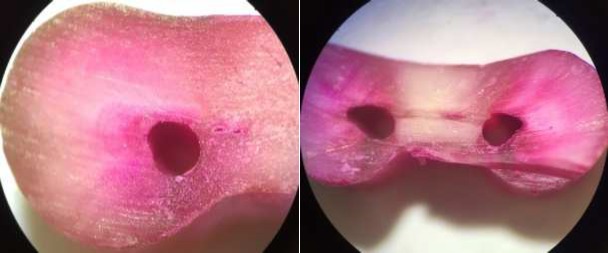
Samples instrumented with Neolix system; *A)* Absence of crack, *B)* Presence of crack

There are also studies reporting that single-file systems can cause less dentinal damage than multi-file systems and it can be due to more manipulations in the canal that leads to more stress concentration [25, 26]. Different methodologies can be a reason for this disagreement. Initial use of hand files (#20 or 25) can provide more space for the rotary single-file instrument. Recently, a #20 single-file was added to Neolix system that can decrease the risk of crack formation when used before # 25.

The risk of micro crack formation by use of the ProTaper system has been evaluated in several studies. Some studies showed high percentage (about 50%) [25, 27]. Some others, including the current study (13.3%), showed less micro cracks in the ProTaper system (ranging from 10% to 30%) [28-31]. Also, a micro-computed tomography (CT) study done by De-Deus *et al.* [32] reported that ProTaper Universal system did not cause any new dentinal defects. According to burklein *et al.* [28], full sequence rotary ProTaper system caused significantly fewer micro cracks than reciprocating files. In another study done by Liu *et al.* [25], ProTaper full sequence rotary system caused cracks in 50% of teeth, whereas reciprocating movement caused micro cracks in only 5% of samples. 

Differences in apical size and taper could cause the conflicting results [33].According to Kim *et al.* [34], there is a relationship between dentinal micro crack formation and instrument design. In the present study, the cross-sectional design varied from non-homothetic rectangular in Neolix group, to flat-sided (D-shaped) in SafeSider system and convex triangular cross-section in ProTaper group. Triangular or modified triangular design of ProTaper cross-section decreases the cutting efficiency and provides less space for dentine chips, thus generating stresses on root canal walls [18]. Our study showed that micro cracks mostly formed in the coronal third. According to Versluis *et al.* [35], stresses generated 1 mm short of the apical foramen were one-third of the stresses in the coronal section. This could be the result of increased taper of the instrument toward the coronal dentinal walls. In the present study, only one complete fracture in samples of ProTaper group was seen. It seems that it may not be purely the result of instrumentation and some other factors probably play a role in this respect. According to Wilcox *et al. *[33], and Shemesh *et al. *[8], the fracture rate varies the current study, obturation and retreatment were not assessed, and this may be the reason for low fracture rate. In the present study, there was no significant difference between SafeSider (reciprocating hybrid system, NiTi and stainless-steel) and ProTaper (rotary NiTi system) group. However, micro cracks occurred in both groups, but with a higher rate in SafeSider system. In the study by Ceyhanli *et al.* [36], no significant difference was reported between ProTaper and SafeSider systems, but micro cracks had a higher frequency in ProTaper group. The difference between our results and theirs may be due to different methodologies. Ceyhanli *et al.* [36] used file sizes 08, 10, 15, 20, 25, 30 and 35 (2% taper, stainless-steel) in the SafeSider group and finally finished by size 30 (4% taper, NiTi). 

**Figure 4:**
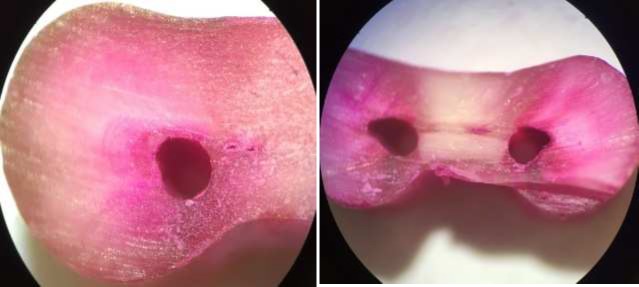
Samples instrumented with SafeSider system (tear drop shaped); *A)* Absence of crack, *B)* Presence of crack

In our study, preparation was done by a master apical file size 25 (2% taper, stainless-steel) and ultimate shaping was done with size 25 (6% and 8% taper, NiTi) and the same standardization was performed among all three groups. The D-shaped and flat-sided design of SafeSider instruments have improved their elasticity [37]. According to the manufacturer, SafeSider files have 16 flutes compared to 24 flutes for files in ProTaper and Neolix groups. It causes less binding with canal walls and decreases fatigue resistance, file fracture or micro crack formation, according to manufacturer [17].

Reciprocating movement has advantages such as more centered position of the instrument in root canal [38]. This movement provides continuous release of the instrument, while engaging the inner surface of the canal wall by repeating the clockwise and counter-clockwise rotation [39]. Blades of the instrument lose contact with dentinal wall during clockwise motion [40]. Furthermore, flexural and torsional forces on dentinal walls may decrease and reduce the screwing effect of the instrument and cause a reduction in dentinal crack formation [41, 42]. Burklein and Schafer [28] reported that reciprocating movement may increase torsional forces by pushing debris toward the root apex. 

Some studies reported more dentinal defects in rotational movement than reciprocal motion [25], while some others support the opposite idea [28]. Also, kinematics had no significant effect on crack formation [43]. Different methodologies can be the reason for this disagreement [25, 28]. According to our study, various canal shapes may be achieved after preparation with different systems. Unlike Neolix that seems to create more symmetric and regular canal cross-sections, SafeSider system seems to make irregular and asymmetric shapes. Ceyhanli *et al. *[44], also found that ProTaper rotary system created better canal shapes than SafeSider system [44]. Yoldas *et al.* [29], also showed that NiTi instruments produced a round cross-section, while another group made a teardrop-shaped cross-section, the same as the initial anatomy of the canal. Craze lines are defects not relevant directly to the pulp space or root canal wall. In the current study, only 2 craze lines were found and they were in Neolix group. Yet, it is not fully known that this may be the result of stress production during canal preparation exceeding the tensile strength of the collagen matrix [33]. In the present study, acrylic blocks and silicon impression material were used to simulate periodontal ligament as a major stress absorber; this was done based on a previous study and can affect the results (19).

Despite most *in vitro* studies that used single-rooted teeth (18, 21-26) for determining the risk of micro crack formation, the current study used both mesial canals of mandibular first molars for instrumentation. This sample selection may also increase the rate of micro cracks.

One of the limitations of the current study was difference in dentin thickness of teeth although we used only mandibular first molars. Also, we could not detect pre-existing defects by our methodology (sectioning and observation under stereomicroscope). Micro-CT has a higher resolution and may be more accurate for detection of dentinal defects in comparison with a stereomicroscope. However, according to Ceyhanli *et al.* [45], image superimpositions did not show a perfect match for pre- and post-instrumentation images. Micro-CT makes hundreds of slices, which are not easy to assess and also some micro cracks may be overlooked.

**Table 1:** Number and percentage of dentinal defects. Only the difference between A and B was statistically significant

	**3 mm**	**6 mm**	**9 mm**	**Total**
**Control**	0(0%)^a^	0(0%)^a^	0(0%)^a^	0(0%)^a *^
**ProTaper**	0(0%)^a^	1(6.7%)^a^	1(6.7%)^a^	2(13.3%)^ab^
**Neolix**	3(20%)^a^	0(0%)^a^	3(20%)^a^	6(40%)^b *^
**SafeSider**	1(6.7%)^a^	1(6.7%)^a^	2(13.3%)^a^	4(26.7%)^ab^
***P value***	0.179	1.000	0.486	0.033

## Conclusion

Neolix rotary single-file system caused more dentinal cracks compared to unprepared roots. Except for the control group, all the instrumentation systems increased the number of micro cracks.

## References

[1] Torabinejad M, Walton RE (2009). Principles and practice of endodontics.

[2] Schilder H (1974). Cleaning and shaping the root canal. Dent Clin North Am.

[3] Singh S, Nigam N (2010). Comparative evaluation of surface characteristics of dentinal walls with and without using plastic finishing file. J Conserv Dent.

[4] Bane K, Faye B, Sarr M, Niang SO, Ndiaye D, Machtou P (2015). Root canal shaping by single-file systems and rotary instruments: a laboratory study. Iran Endod J.

[5] Bergmans L, Van Cleynenbreugel J, Beullens M, Wevers M, Van Meerbeek B, Lambrechts P (2002). Smooth flexible versus active tapered shaft design using NiTi rotary instruments. Int Endod J.

[6] Blum JY, Machtou P, Ruddle C, Micallef JP (2003). Analysis of mechanical preparations in extracted teeth using ProTaper rotary instruments: value of the safety quotient. J Endod.

[7] Blum J, Machtou P, Ruddle C, Micallef J (2003). Analysis of mechanical preparations in extracted teeth using ProTaper rotary instruments: value of the safety quotient. J Endod.

[8] Shemesh H, Bier CA, Wu MK, Tanomaru-Filho M, Wesselink PR (2009). The effects of canal preparation and filling on the incidence of dentinal defects. Int Endod J.

[9] Jalali S, Eftekhar B, Paymanpour P, Yazdizadeh M, Jafarzadeh M (2015). Effects of Reciproc, Mtwo and ProTaper Instruments on Formation of Root Fracture. Iran Endod J.

[10] Shemesh H, Bier C, Wu MK, Tanomaru‐Filho M, Wesselink P (2009). The effects of canal preparation and filling on the incidence of dentinal defects. Int Endod J.

[11] Bier CA, Shemesh H, Tanomaru-Filho M, Wesselink PR, Wu MK (2009). The ability of different nickel-titanium rotary instruments to induce dentinal damage during canal preparation. J Endod.

[12] Varela-Patiño P, Ibañez-Párraga A, Rivas-Mundiña B, Cantatore G, Otero XL, Martin-Biedma B (2010). Alternating versus continuous rotation: a comparative study of the effect on instrument life. J Endod.

[13] Varela-Patino P, Ibanez-Parraga A, Rivas-Mundina B, Cantatore G, Otero XL, Martin-Biedma B (2010). Alternating versus continuous rotation: a comparative study of the effect on instrument life. J Endod.

[14] De-Deus G, Brandao MC, Barino B, Di Giorgi K, Fidel RA, Luna AS (2010). Assessment of apically extruded debris produced by the single-file ProTaper F2 technique under reciprocating movement. Oral Surg Oral Med Oral Pathol Oral Radiol Endod.

[15] Webber J, Machtou P, Pertot W, Kuttler S, Ruddle C, West J (2011). The WaveOne single-file reciprocating system. Roots.

[16] Hargreaves KM, Berman LH (2015). Cohen's pathways of the pulp.

[17] https://www.neolix.eu/en/6-files.

[18] Burklein S, Hinschitza K, Dammaschke T, Schafer E (2012). Shaping ability and cleaning effectiveness of two single-file systems in severely curved root canals of extracted teeth: Reciproc and WaveOne versus Mtwo and ProTaper. Int Endod J.

[19] http://edsdental.com/safesiders.htm.

[20] Schneider SW (1971). A comparison of canal preparations in straight and curved root canals. Oral Surg Oral Med Oral Pathol.

[21] Zhang R, Hu T (2010). Root canal curvature. Int Endod J.

[22] De-Deus G, Silva EJNL, Marins J, Souza E, de Almeida Neves A, Belladonna FG, Alves H, Lopes RT, Versiani MA (2014). Lack of causal relationship between dentinal microcracks and root canal preparation with reciprocation systems. J Endod.

[23] Gavini G, Caldeira CL, Akisue E, Candeiro GT, Kawakami DA (2012). Resistance to flexural fatigue of Reciproc R25 files under continuous rotation and reciprocating movement. J Endod.

[24] Priya NT, Chandrasekhar V, Anita S, Tummala M, Raj TB, Badami V, Kumar P, Soujanya E (2014). "Dentinal microcracks after root canal preparation" a comparative evaluation with hand, rotary and reciprocating instrumentation. J Clin Diagn Res.

[25] Liu R, Hou BX, Wesselink PR, Wu M-K, Shemesh H (2013). The incidence of root microcracks caused by 3 different single-file systems versus the ProTaper system. J Endod.

[26] Shemesh H, Roeleveld AC, Wesselink PR, Wu MK (2011). Damage to root dentin during retreatment procedures. J Endod.

[27] Kansal R, Rajput A, Talwar S, Roongta R, Verma M (2014). Assessment of dentinal damage during canal preparation using reciprocating and rotary files. J Endod.

[28] Bürklein S, Tsotsis P, Schäfer E (2013). Incidence of dentinal defects after root canal preparation: reciprocating versus rotary instrumentation. J Endod.

[29] Yoldas O, Yilmaz S, Atakan G, Kuden C, Kasan Z (2012). Dentinal microcrack formation during root canal preparations by different NiTi rotary instruments and the self-adjusting file. J Endod.

[30] Karataş E, Gündüz H, Kırıcı D, Arslan H (2015). Incidence of dentinal cracks after root canal preparation with ProTaper Gold, Profile Vortex, F360, Reciproc and ProTaper Universal instruments. Int Endod J.

[31] Monga P, Bajaj N, Mahajan P, Garg S (2015). Comparison of incidence of dentinal defects after root canal preparation with continuous rotation and reciprocating instrumentation. Singapore Dent J..

[32] De-Deus G, Belladonna FG, Marins JR, Silva EJNL, Neves AdA, Souza EM, Machado AdC, Lopes RT, Versiani MA (2016). On the causality between dentinal defects and root canal preparation: A micro-CT assessment. Braz Dent J.

[33] Wilcox LR, Roskelley C, Sutton T (1997). The relationship of root canal enlargement to finger-spreader induced vertical root fracture. J Endod.

[34] Kim H-C, Lee M-H, Yum J, Versluis A, Lee C-J, Kim B-M (2010). Potential relationship between design of nickel-titanium rotary instruments and vertical root fracture. J Endod.

[35] Versluis A, Messer HH, Pintado MR (2006). Changes in compaction stress distributions in roots resulting from canal preparation. Int Endod J.

[36] Ceyhanli KT, Erdilek N, Tatar I, Celik D (2016). Comparison of ProTaper, RaCe and Safesider instruments in the induction of dentinal microcracks: a micro-CT study. Int Endod J.

[37] Musikant BL, Cohen BI, Deutsch AS (2004). Comparison instrumentation time of conventional reamers and files versus a new, noninterrupted, flat-sided design. J Endod.

[38] Franco V, Fabiani C, Taschieri S, Malentacca A, Bortolin M, Del Fabbro M (2011). Investigation on the shaping ability of nickel-titanium files when used with a reciprocating motion. J Endod.

[39] Yared G (2008). Canal preparation using only one Ni‐Ti rotary instrument: preliminary observations. Int Endod J.

[40] Prichard J (2012). Rotation or reciprocation: a contemporary look at NiTi instruments? Br Dent J.

[41] Wei X, Hu B, Peng H, Tang M, Song J (2017). The incidence of dentinal cracks during root canal preparations with reciprocating single-file and rotary-file systems: A meta-analysis. Dent Mater J.

[42] Berutti E, Chiandussi G, Paolino DS, Scotti N, Cantatore G, Castellucci A, Pasqualini D (2012). Canal shaping with WaveOne Primary reciprocating files and ProTaper system: a comparative study. J Endod.

[43] Ustun Y, Aslan T, Sagsen B, Kesim B (2015). The effects of different nickel-titanium instruments on dentinal microcrack formations during root canal preparation. Eur J Dent.

[44] Ceyhanli KT, Erdilek N, Tatar I, Çetintav B (2014). Comparative micro‐computed tomography evaluation of apical root canal transportation with the use of ProTaper, RaCe and Safesider systems in human teeth. Aust Endod J.

[45] Ceyhanli KT, Erdilek N, Tatar I, Celik D (2016). Response to comments on to our published article entitled: 'Comparison of ProTaper, RaCe and Safesider instruments in the induction of dentinal microcracks: a micro-CT study'. Int Endod J.

